# Comparing the SF-12 and SF-36 health status questionnaires in patients with and without obesity

**DOI:** 10.1186/1477-7525-6-11

**Published:** 2008-01-30

**Authors:** Christina C Wee, Roger B Davis, Mary Beth Hamel

**Affiliations:** 1 Division of General Medicine and Primary Care, Department of Medicine, Beth Israel Deaconess Medical Center, Harvard Medical School, Boston, Massachusetts, USA

## Abstract

**Objective:**

To assess how well the SF-36, a well-validated generic quality of life (QOL) instrument, compares with its shorter adaptation, the SF-12, in capturing differences in QOL among patients with and without obesity.

**Methods:**

We compared the correlation between the physical (PCS) and mental (MCS) component summary measures of the SF-12 and SF-36 among 356 primary care patients using Pearson coefficients (r) and conducted linear regression models to see how these summary measures captures the variation across BMI. We used model R^2 ^to assess qualitatively how well each measure explained the variation across BMI.

**Results:**

Correlations between SF-12 and SF-36 were higher for the PCS in obese (r = 0.89) compared to overweight (r = 0.73) and normal weight patients (r = 0.75), p < 0.001, but were similar for the MCS across BMI. Compared to normal weight patients, obese patients scored 8.8 points lower on the PCS-12 and 5.7 points lower on the PCS-36 after adjustment for age, sex, and race; the model R^2 ^was higher with PCS-12 (R^2 ^= 0.22) than with PCS-36 (R^2 ^= 0.16). BMI was not significantly associated with either the MCS-12 or MCS-36.

**Conclusion:**

The SF-12 correlated highly with SF-36 in obese and non-obese patients and appeared to be a better measure of differences in QOL associated with BMI.

## Background

In addition to its etiologic role in many common medical conditions, obesity has profound adverse physical, social, and economic consequences that can negatively affect quality of life, an increasingly important outcome considered by patients, clinicians, and policymakers alike. As a result, quality of life has become an important endpoint assessed in studies of obesity and weight loss interventions.

One of the most straightforward ways of measuring quality of life (QOL) is through the use of health status measures where patients are asked to rate different aspects of their life. Perhaps the most commonly used measure in QOL research is the Short-Form 36 or SF-36, a generic measure developed and validated in the Medical Outcomes Study to assess important QOL domains relevant to patients suffering from a wide range of medical conditions [1]. The SF-36 consists of eight QOL domains that comprise two summary measures – the physical component summary and the mental component summary. One of the major advantages of using the SF-36 in studies of obesity is that it allows for QOL scores to be compared to scores in other common diseases. However, because the SF-36 was not originally designed to measure important QOL domains specific to obesity, a number of studies have found the SF-36, particularly the mental component summary, to be relatively insensitive to variations in body weight cross-sectionally or to changes in weight over time [2-4]. As a consequence, obesity-specific health measures such as the Impact of Weight on Quality of Life and the Moorehead-Ardelt QOL Questionnaire have been developed to address these limitations [5-7]. However, because of their specificity, these instruments cannot be used to compare the QOL impact of obesity and changes in weight with the QOL impact of other diseases. Thus, studies of obesity may need to include a combination of different instruments especially when QOL is a primary outcome. This approach, however, can pose a high burden on participants and may affect study participation rates and the cost of conducting research.

To address the considerable burden placed on respondents and investigators generically, Ware and colleagues developed a substantially shorter questionnaire, the SF-12, which utilized a reduced number of items from 36 to 12. The SF-12 can be completed by most participants in less than a third of the usual time needed to complete the SF-36 [8]. Ware found the two instruments to be highly correlated and both the physical and mental component summary measures in the SF-12 explained about 90% of the variation in the same summary measures of the SF-36 [8,9]. Subsequent studies [10-12] comparing the two instruments have suggested varying results depending on the disease or health condition of interest. While the two measures performed similarly in studies of patients with cardiac and pulmonary conditions, the mental component summary score correlated less well in studies on arthritis [10-12]. Whether the SF-12 and SF-36 can be used interchangeably for studies in obesity is unclear.

In this context, we compared the SF-12 and the SF-36 in a cross-sectional sample of primary care patients of varying body weights to examine the correlation between the two instruments and their performance in measuring differences in QOL among patients with and without obesity.

## Methods

### Study Sample

We conducted a 25-minute telephone interview of a random sample of 366 patients seen at a large hospital-based primary care practice in Boston between November 2001 and June 2003. The goal of the study was to describe the quality of life and weight-related health behaviour of primary care patients and to quantify the value they placed on different levels of weight loss. Eligible patients were 18 years and older, English-speaking, and free from terminal or serious illness that would prevent them from participating. Details on subject recruitment and sampling have been published elsewhere [13,14]. The response rate was 60%. This present study includes the 356 subjects with complete information on quality of life measures. The study was approved by the Institutional Review Board (IRB) at Beth Israel Deaconess Medical Center (#2001-P-000119). Verbal informed consent was obtained for publication from the participants and/or their relatives as approved by the IRB.

### Data Collection and Measures

The telephone interview was administered by trained interviewers and ascertained information such as patient demographics, height, weight, comorbid illness, and quality of life. We calculated body mass index (BMI) from self-reported height and weight and categorized respondents as normal weight, overweight, and obese according to standard guidelines [15]. Quality of life (QOL) was assessed using the Short-Form 36 or SF-36, a generic health status instrument with 36 items comprising eight subscales – physical functioning, role functioning (physical and emotional), bodily pain, general health, vitality, social functioning, and mental health. Using standard methods [1], we calculated the two summary measures that comprise the SF-36: the physical component summary (PCS-36) and the mental component summary (MCS-36). Scores of each subscale are calculated based on the response to individual items comprising that subscale; the subscales are then standardized using a z-score transformation and aggregated to estimate the aggregated physical and mental summary scores. While all eight subscales contribute to both summary scores, the physical component summary score is more heavily weighted by the physical functioning subscale followed by physical role function, bodily pain, general health and vitality subscales whereas the mental component summary score is driven by the mental health subscale followed by emotional role functioning, social functioning and the vitality subscales. We also calculated SF-12 component summary scores (PCS-12 and MCS-12) using SF-12 items embedded in the SF-36 [1]. This approach has been shown to be equivalent to calculating SF-12 derived from the SF-12 as a standalone questionnaire [8,16]. All four summary scores range from 0–100 where higher scores indicated better QOL.

### Data Analysis

For each respondent, we calculated the PCS-12, MCS-12, PCS-36, and MCS-36 score based on responses to the relevant items. We then calculate the mean PCS-12, MCS-12, PCS-36, and MCS-36 score for the overall study sample. To examine how well the PCS-12 correlated with the PCS-36 and how well the MCS-12 correlated with the MCS-36 among different BMI groups, we determined the Pearson correlation coefficient to describe the correlation between the respective measures stratified by BMI. To determine whether these correlations between the two PCS and MCS measures were significantly different among BMI groups, we tested the homogeneity of correlations across BMI categories using the method described by Zar, et al [17]. We then used linear regression models to explain how the PCS-12 and MCS-12 scores varied relative to PCS-36 and MCS-36 scores, respectively. We tested for an interaction between the SF-12 summary scores and age, sex, race, and BMI to examine whether the relationship between PCS-12 and PCS-36 and between MCS-12 and MCS-36 might vary depending on these factors.

To examine how well the summary measures from the SF-12 and SF-36 performed in distinguishing respondents of varying BMI, we first tested the unadjusted association between BMI category and the four QOL summary measures using the Wilcoxon Rank Sum test. We then used linear regression modelling to examine the relationship between BMI and these four QOL summary measures after adjusting for age, sex, and race. We used the R-square of each model (model R^2^) to assess the performance of the component summary measures of the SF-12 and SF-36 in discriminating among patients of different BMI category; the higher the model R^2^, the better the summary score is able to explain variations in quality of life associated with BMI.

## Results

Of 356 respondents, the mean age was 48.9 ± 0.83 years (range 19–90) and the mean BMI was 27.8 ± 0.38 kg/m^2^. Table 1 presents the additional characteristics of our study sample and mean quality of life summary scores.

**Table 1:** Study Population Characteristics (n = 356)

	n (%)
Age, y	
19–29	44 (12)
30–49	158 (43)
50–64	101 (28)
65 and older	62 (17)
Weight Category	
Normal Weight (18.5 to 24.9 kg/m^2^)	139 (39)
Overweight	117 (33)
Obese	98 (28)
Sex	
Men	124 (35)
Women	232 (65)
Race/Ethnicity	
White	245 (69)
Black	71 (20)
Hispanic	15 (4)
Asian	11 (3)
Other	12 (3)
Summary scores, mean (SD)	
PCS-36	
Overall	45.2 (8.0)
Normal weight	47.9 (6.6)
Overweight	45.4 (7.5)
Obese	41.3 (8.8)
PCS-12	
Overall	49.7 (10.0)
Normal weight	54.1 (7.7)
Overweight	49.3 (9.2)
Obese	44.0 (10.8)
MCS-36	
Overall	52.4 (10.1)
Normal weight	53.1 (9.4)
Overweight	52.7 (11.5)
Obese	51.0 (12.0)
MCS-12	
Overall	55.3 (10.7)
Normal weight	55.7 (9.5)
Overweight	55.8 (10.8)
Obese	54.0 (12.0)

**Figure 1:**
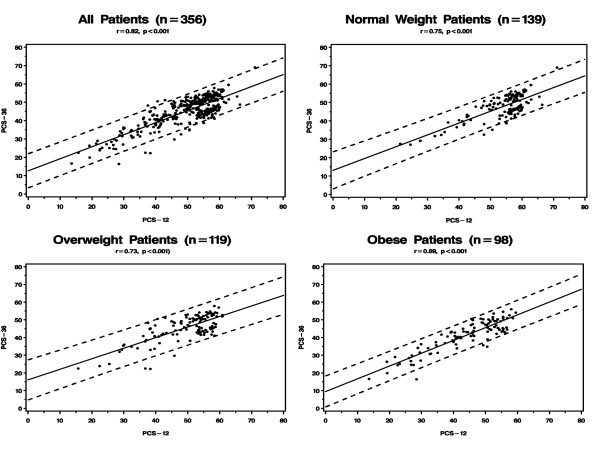
Correlation Between the Physical Component Summary (PCS) Measures of the SF-12 and SF-36.

**Figure 2:**
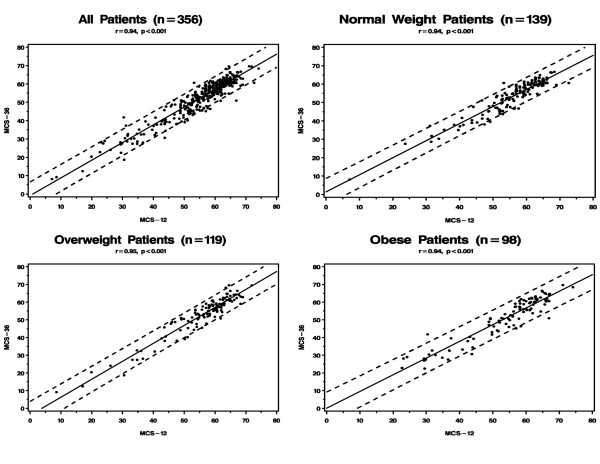
Correlation Between the Mental Component Summary (MCS) Measures of the SF-12 and SF-36.

Figures 1 and 2 presents the correlation between PCS-12 and PCS-36 and between MCS-12 and MCS-36 for the entire sample and by BMI group. The Pearson correlation was 0.82 for the physical component summary scores and 0.95 for the mental component summary scores overall. The correlation between PCS-12 and PCS-36 varied significantly according to BMI with the highest correlation observed in patients who were obese (p < 0.001 for differences in correlation among the three BMI groups); the correlations between MCS-12 and MCS-36 were very similar across BMI.

Using linear regression, we estimated that PCS-12 and MCS-12 can be derived from the respective PCS-36 and MCS-36 using the following equations:

PCS-36 score = 0.66 (PCS-12 score) + 12.63   model R^2 ^= 0.67

MCS-36 score = 0.96(MCS-12) - 0.82   model R^2 ^= 0.89

The relationship between the two sets of summary measures did not vary significantly by age, sex, race or BMI, although the interaction between PCS-12 and BMI category approached statistical significance (p = 0.08).

Table 1 also shows the mean QOL scores across BMI category. Before adjustment, BMI was significantly associated with mean PCS-12 and PCS-36 scores but not for MCS-12 and MCS-36 scores (Table 1). After adjustment for age, sex, and race, obese patients scored 5.7 points lower than normal weight patients on the PCS-36, whereas overweight patients did not score significantly differently compared to normal weight patients (Table 2), although the overall trend between higher BMI category and lower score was statistically significant (p < 0.001). In contrast, obese patients scored 8.8 points lower and overweight patients scored 4.0 points lower on the PCS-12 than normal weight patients (p-trend < 0.001). The model R^2 ^was higher for BMI and PCS-12 (R^2 ^= 0.22) than for BMI and PCS-36 (R^2 ^= 0.16), suggesting that when compared to PCS-36, PCS-12 is better able to explain the variation in quality of life among patients with different BMI. Body mass index was not significantly associated with either MCS-12 or MCS-36 although there was more of a suggested trend between BMI group and MCS-36 (p = 0.05) than with MCS-12 (p = 0.10). The model R^2 ^for both MCS-12 (R^2 ^= 0.03) and MCS-36 (R^2 ^= 0.04) were extremely low.

**Table 2:** Difference in SF-12 and SF-36 component summary scores for overweight and obesity compared to normal weight after adjustment for age, race, and sex.

	PCS-12*	PCS-36*	**MCS-12**	**MCS-36**
Normal weight (BMI 18.5–<25 kg/m^2^)	Reference	Reference	Reference	Reference
Overweight (BMI >25 kg/m^2^)	-4.0	-1.6	-0.4	-1.0
Obese (BMI >30 kg/m^2^)	-8.8	-5.7	-2.6	-3.1

## Discussion

Our study suggests that the SF-12 and SF-36 correlates very highly regardless of BMI but especially among patients with obesity. Moreover, the physical component summary measure of the SF-12 (PCS-12) appeared to better explain differences in quality of life (QOL) among patients with different BMI than the PCS-36. Mental component summary (MCS) scores on both the SF-12 and SF-36 did not vary significantly by BMI.

Previous studies comparing the SF-12 and SF-36 in patients with specific diseases or health conditions have generally found moderate to high correlations between the physical and mental component summary measures of both instruments [8,9]. Ware et al reported high correlations between the SF-12 and SF-36 among patients in the Medical Outcomes Study [8,9]. Another longitudinal study of over 2400 patients with heart disease found that the Pearson correlations between both summary measures of the SF-12 and SF-36 ranged from 0.94 to 0.96 [10]. Moreover, all measures were sensitive to changes over time. A smaller study of three groups of patients about to undergo treatment for congestive heart failure, obstructive sleep apnea, and surgical hernia repair respectively found that the summary measures for both SF-12 and SF-36 across BMI performed similarly before and after treatment [11].

Our findings show that BMI was strongly associated with the physical component summary measure but not the mental component summary measure of the SF-36 is supported by prior work [18,19]. Using data from the Medical Outcomes Study, Katz and colleagues found that compared to normal weight patients, overweight and obese patients had larger decrements in subscales, with the largest contribution to the physical component summary measure such as physical function, physical role function, general health, and vitality than the subscales that represent mental, emotional and social functioning [18]. Subsequent studies [19] have largely confirmed these findings. Fewer studies have used the SF-12 to examine QOL differences across BMI, but one survey of primary care patients by Finkelstein et al. found that PCS-12 decreased consistently with higher BMI above the normal weight range but the relationship between BMI and MCS-12 was curvilinear: persons who were overweight but not obese had lower MCS-12 scores than those who were normal weight or obese [20]. To our knowledge, prior studies have not directly compared the SF-12 and SF-36 within the same study population.

Our study demonstrates high correlations between SF-12 and SF-36 in both the physical and mental component summary measures regardless of BMI. While we expected reasonably high correlations since the SF-12 is embedded in the SF-36, we found that correlations between the SF-12 and SF-36 for the physical component scales were actually highest among patients who were obese. We also found that PCS-12 performed better than the SF-36 in explaining variations in QOL across BMI. Since the MCS-36 has been shown in this and other studies to be relatively insensitive to differences in BMI, using the MCS-12 instead of MCS-36 is unlikely to produce poorer measurement of obesity-related QOL. Taken together, these findings suggest that the SF-12 may be an adequate substitute for the SF-36 in studies on obesity with two caveats. First, our study was cross-sectional and whether the SF-12 would be as sensitive as the SF-36 to changes in weight over time is unclear, although our finding of greater variability across BMI for the PCS-12 as compared to the PCS-36 is reassuring. Second, because of the brevity of the SF-12 instrument, it is not possible to obtain reliable information for each of the eight domains or subscales that comprise the overall SF-12 so that one would not be able to draw conclusions about specific domains that contribute to QOL as measured by the two component summary measures.

Finally, our findings must also be interpreted in the context of our study's limitations. We sampled patients from one large academic primary care practice in Boston where the BMI distribution of the population closely mirrors the general US population. Whether our results would apply to patients with more severe obesity, those actively seeking weight treatments, or those from other geographic regions are unclear. In addition, our BMI values were calculated from self-reported height and weight and studies suggest that some respondents, especially women, tend to overestimate their height and underestimate weight leading to underestimation of BMI [21,22]; whereas others, including men and older adults, tend to over report weight [23]. These misclassifications will tend to bias findings towards detecting no difference and might underestimate potential differences observed across BMI. Our multivariable models did adjust for these demographics factors. Finally, we administered our survey via telephone in order to ensure a random sample and to optimize participation. While this approach minimizes barriers to survey participation such as low literacy, scoring norms may differ between mail versus telephone administered instruments [24]; however, findings related to comparisons made between SF-12 and SF-36 in our study are likely still valid.

## Conclusion

Our study suggests that the SF-12 correlates highly with the SF-36 in patients of all BMI groups and appears to perform at least as well as the SF-36 in cross-sectional settings; hence, using the SF-12 in place of the SF-36 may be appropriate, especially when other more obesity-specific QOL measures are being used and when respondent burden is a major concern. Future studies should validate our findings longitudinally and in more diverse populations.

## Competing interests

The author(s) declare that they have no competing interests.

## Authors' contributions

CW conceived of the study, conducted the study, analyzed and interpreted the data and drafted the manuscript. RD and MBH contributed to the design and interpretation of the analysis. All authors read and critically revised and approved the final manuscript.
